# Refractory ascites as a presenting feature of extramedullary plasmacytoma in an end-stage renal disease patient with HIV infection

**DOI:** 10.5414/CNCS109560

**Published:** 2019-02-10

**Authors:** Karim Soliman, Johann Herberth, Tibor Fülöp, Angie Duong, Rachel L. Sturdivant

**Affiliations:** 1 Department of Medicine, Division of Nephrology, Medical University of South Carolina,; 2 Medical Services, Ralph H. Johnson VA Medical Center,; 3 Department of Pathology, Medical University of South Carolina, Charleston, SC, USA, and; 4 Department of Medicine, Division of Nephrology, Cairo University, Egypt

**Keywords:** ascites, end-stage renal disease, extramedullary plasmacytoma, dialysis, malignancy, peritoneal dialysis

## Abstract

Refractory ascites as the only presenting feature of an extramedullary plasmacytoma complicating end-stage renal disease and HIV infection has not been described yet. We describe a case of a 39-year-old female with HIV-associated nephropathy manifesting with ascites formation after transition from peritoneal dialysis (PD) to hemodialysis (HD). Earlier on, she received cycler-assisted PD for 5 years uneventfully. A few weeks after HD transition, a striking refractory ascites developed requiring multiple paracenteses (5 – 7 L every second week). Serum protein electrophoresis showed hypoalbuminemia with only small amount of monoclonal IgG-κ at 0.30 g/dL. Serum immunofixation electrophoresis showed polyclonal immunoglobulins with polyclonal light chains. Both κ and λ light chains were increased, at 66.86 mg/dL (reference range: 0.33 – 1.94) and 18.55 mg/dL (reference range: 0.57 – 2.63), respectively, with a ratio of 3.6 (reference range: 0.26 – 1.65). However, an ascitic fluid analysis showed a marked increase in plasma cells with a κ : λ ratio greater than 5 : 1. Omental biopsy confirmed κ-restricted plasma cells. Multiple myeloma work-up with skeletal survey showed no evidence of focal osseous lesions, while bone marrow aspiration and biopsy also remained unremarkable. Accordingly, the diagnosis of omental extramedullary plasmacytoma with malignant ascites was confirmed. Conversion from PD to HD may unmask an underlying pathology favoring ascites formation.

## Introduction 

Immunocompromised patients with end-stage renal disease (ESRD) and HIV infection are at an increased risk for developing various kinds of malignancies, including plasma cell dyscrasia. Ascites is rarely encountered as a presenting feature of the extramedullary formation of plasmacytoma [1] and represents only 3% of all plasma cell dyscrasias. Solitary plasmacytoma can be divided into two groups: solitary bone plasmacytoma and solitary extramedullary plasmacytoma (EMP). The most common site for EMP is the upper respiratory tract, but a gastrointestinal and omental involvement is rare [2]. Cytomorphology and immunophenotyping of ascitic fluid can easily diagnose omental EMP even without omental biopsy [3]. EMP progresses to multiple myeloma in 11 – 30% of patients within 10 years [4]. Refractory ascites as the only presenting feature of extramedullary plasmacytoma in an ESRD patient with HIV on dialysis has not been reported before. 

## Case presentation 

A 39-year-old female presented with complaints of generalized abdominal distension, weight loss, swelling of legs, and muscle weakness extant for several weeks. Her past medical history was significant for a well-controlled HIV infection of 6 years on highly active anti-retroviral therapy, hypertension, ESRD secondary to HIV-associated nephropathy, and secondary hyperparathyroidism. She was a non-smoker and had no history of alcohol or drug abuse. Her initial renal biopsy revealed collapsing focal glomerular sclerosis, microcystic dilation of renal tubules, lymphocytic interstitial infiltrates, and interstitial fibrosis. She received chronic cycler-assisted peritoneal dialysis (PD) for 5 years and was well controlled clinically regarding uremic and volume status with no abnormalities regarding color, viscosity, or volume of exchanges. Subsequently, she was transitioned to intermittent hemodialysis (HD) due to personal preferences. A few weeks after the transition to intermittent HD, ascites of unclear etiology developed, ultimately requiring repeated large-volume paracentesis with ~ 5 – 7 L ascitic fluid removals every other week. 

There were no associated symptoms of coughing, palpitations, or shortness of breath. She was chronically anuric. Vital signs showed blood pressure of 160/80 mmHg, heart rate 74 beats/min (regular), respiratory rate 16/min and temperature 36.9 °C. Oxygen saturation was 98% on ambient room air. Her physical examination was remarkable for moderate ascites and edema of the lower extremities. Otherwise neurological, respiratory, and cardiovascular examinations were normal without organomegaly and without clinical stigmata or symptoms of chronic liver disease or heart failure. 

Laboratory investigations showed normocytic, normochromic anemia with a hemoglobin of 10 g/dL and uncorrected serum calcium of 8.6 mg/dL. Other labs were as follows: CD4 T-cell count: 600/mm^3^, HIV viral load: 986 copies/mL (reference: < 40 copies/mL), serum creatinine: 10.3 mg/dL, aspartate aminotransferase: 13 U/L (normal range: 5 – 34 U/L), alanine aminotransferase: 17 U/L (5 – 45 U/L), bilirubin: 0.4 mg/dL, prothrombin time INR: 1.01, serum albumin: 2.3 mg/dL, serum amylase: 105 IU/L (27 – 130 U/L), serum lipase: 40 IU/L (8 – 78 U/L). Her tumor antigens (Ag) were within reference range for cancer antigen (CA) 15-3 and CA19-9. CA 125 levels were repeatedly elevated (197 – 416 U/mL (normal: < 34.9 U/mL)), but no further follow-up was obtained. Analysis of the ascites fluid showed an albumin level of 1.5 g/dL and protein of 3.5 g/dL. The serum-to-ascites albumin gradient was < 1.1 excluding portal hypertension as a cause of ascites. The culture of peritoneal fluid was negative for acid-fast bacilli. There were no fungi seen by calcofluor white stain. *Histoplasma* Ag and *Aspergillus* Ag were negative. The pancreas was reported normal on computed tomography. Her β-2 microglobulin was elevated to 47.30 mg/L (normal: 0.80 – 2.3). Serum protein electrophoresis showed hypoalbuminemia and decreased total protein, with minimal monoclonal M-component only (IgG-κ 0.30 g/dL in the γ zone). Immunofixation electrophoresis showed polyclonal immunoglobulins (Ig) with polyclonal light chains. κ and λ light chains were increased to 66.86 mg/dL (normal: 0.33 – 1.94) and 18.55 mg/dL (normal: 0.57 – 2.63), respectively, yielding a ratio of 3.6 (normal: 0.26 – 1.65) (Table 1). Ascitic fluid analysis showed a marked increase in plasma cells with an abnormal κ : λ ratio, i.e., > 5 : 1. 

**Figure 1.**
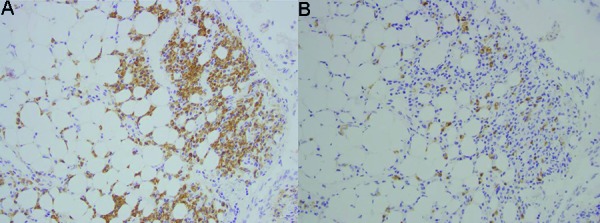
Immunohistochemistry showing κ (A) and λ staining (B).

Liver biopsy was essentially normal, hence an omental biopsy was performed which on immunohistochemistry showed similar findings as the ascitic fluid with predominant κ and faint λ staining (Figure 1). Multiple myeloma work-ups with skeletal surveys revealed no focal osseous lesions, apart from the osseous changes of hyperparathyroidism. Accordingly, the diagnosis of omental EMP with malignant ascites was established. Omental resection and radiation were offered to the patient, but she refused further intervention. Two years of follow-up, she remained stable without any further complication, apart from requiring regular paracenteses. 

## Discussion 

Differential diagnoses of increased plasma cells in the blood include plasma cell neoplasms, such as myeloma, marginal zone lymphoma with plasmacytic differentiation, and lymphoplasmacytic lymphoma. EMP constitutes only 3% of plasma cell tumors. It is defined as either primary (without evidence of co-existing multiple myeloma), or secondary (associated with multiple myeloma). The International Myeloma Working Group defined EMP by the following criteria: (1) no monoclonal Ig in serum or urine; (2) a tumor composed of monoclonal plasma cells in a single extramedullary site; (3) no lesion in the bone marrow; (4) no lesion in the whole-body bone; and (5) no involvement of organs [5]. The most common site for EMP is the upper respiratory tract, including the oropharynx, nasopharynx, larynx, and nasal sinuses. The involvement of the gastrointestinal (GI) tract is rare. In a review of 161 total cases of EMP, only 12 were GI in origin [1], with the small bowel being the most common site affected followed by the stomach and the colon [4, 6, 7, 8]. Gastrointestinal plasmacytoma may present with non-specific symptoms like anorexia, vomiting, weight loss, abdominal pain, and rarely, GI bleeding from ulcerating lesion. Ascites as a presenting feature of plasma cell dyscrasia has rarely been documented in case reports [3, 8, 9]. The differential diagnosis of EMP includes lymphoplasmacytic lymphoma and plasmacytoma differentiated from each other by CD45 and CD20 immunostains [10, 11]. The immunohistochemistry of EMP tumor cells is negative for CD20 and CD45, while in lymphoplasmacytic lymphoma, it is positive for CD20. In plasmacytoma, CD45 is variable, while CD20 is mostly negative. Overall, in a multicenter study on 24 HIV seropositive patients and 60 controls with ascites, HIV-seropositive patients presented similar clinical findings. However, HIV patients had a significantly higher incidence of elevated temperature, lower serum albumin and lower leucocyte count. They also had a higher prevalence of infected ascites than the control subjects (spontaneous bacterial peritonitis in 4 cases, tuberculous ascites in 2, fungal peritonitis and lymphoma in 1) [12]. Interestingly, we have also found an elevated CA 125 level in our patient, which appears to be elevated in ovarian plasmacytoma [13]. Our index case, however, presented with diffuse involvement of the omentum, rather than with isolated ovarian process. However, to our knowledge, this is the first case report of a combined presentation of HIV, ESRD, and EMP. Moreover, our case highlights that conversion from PD to HD may unmask any underlying pathology favoring ascites formation [14]. 

## Conclusion 

We report a case of primary omental plasma cell dyscrasia as a cause of ascites in an HIV patient initially on PD. This clinical finding was only unmasked after the patient switched to HD. Diagnosing EMP as a cause of ascites is challenging in itself, but it becomes even more problematic when an ESRD modality is used that utilizes the peritoneal cavity (in PD) and covers up signs and symptoms of ascites. In light of the improved long-term survival of HIV patients on highly active anti-retroviral therapy, vigilance about HIV-associated comorbid conditions becomes more important. Our case is to raise awareness of illnesses that are associated with ascites in this patient population and potentially treatable illnesses such as EMP. 

## Acknowledgment 

Drs. Fülöp and Herberth are current employees of the United States Veterans Health Administration. However, the views and opinions expressed herewith do not reflect the official views or opinion or endorsed by the United States Veteran Health Administrations. 

We sincerely appreciate the assistance of Mr. Attila Lénárt-Muszka during editing and grammar review. 

## Funding 

This work was not supported by any grant. 

## Conflict of interest 

The authors have no conflict of interest to declare. 

**Table 1.** Trend of abnormal κ : λ ratio spanning 2016 through 2017.

	Reference range & units	3/28/2017	10/31/2016	7/28/2016	7/26/2016	6/26/2016
κ free light chains	0.33 – 1.94 mg/dL	130.8	99.00	45.77	54.66	66.86
λ free light chains	0.57 – 2.63 mg/dL	27.51	20.86	16.58	19.86	18.55
κ : λ free light chains ratio	0.26 – 1.65 ratio	4.74	4.75	2.76	2.75	3.6
